# Melatonin effect on platelet count in patients with liver disease 

**Published:** 2021

**Authors:** Ayda Esmaeili, Mohssen Nassiri Toosi, Mohammad Taher, Jaleh Bayani, Soha Namazi

**Affiliations:** 1 *Hematology, Immune Cell Therapy, and Stem Cell Transplantation Research Center, Clinical Research Institute, Urmia University of Medical Sciences, Urmia, Iran*; 2 *Clinical Pharmacy Department, Faculty of Pharmacy, Urmia University of Medical Sciences, Urmia, Iran*; 3 *Internal Medicine Department, School of Medicine, Tehran University of Medical Sciences (TUMS), Tehran, Iran*; 4 *Liver Transplantation Research Center, Tehran University of Medical Sciences, Tehran, Iran*; 5 *Clinical Pharmacy Department, school of Pharmacy, Tehran University of Medical Sciences (TUMS), Tehran, Iran *

**Keywords:** Platelet, Melatonin, Liver disease, Thrombopoietic properties

## Abstract

**Aim:**

A positive effect of melatonin on platelet count in patients with chronic liver disease is reported in the current study.

**Background:**

Thrombocytopenia occurs when the severity of liver disease is exacerbated. Reduction in the secretion of *thrombopoetin*, as an intrinsic hormone produced mainly by the liver, plays an important role in this complication induced by liver disease.

**Methods:**

This research was a double-blind, cross-over, placebo-controlled pilot study. Patients with liver disease were given two 5-mg pearls of melatonin or a placebo for two weeks, and after a 2-week washout period, their groups were switched. Liver function tests and platelet counts were assessed once at the beginning and once at the end of each phase of the study.

**Results:**

In the current study, 40 patients meeting the eligibility criteria were included. The average platelet count was significantly increased by melatonin in comparison with the placebo (from 175.67±92.84 to 191.10±98.82 vs. from 185.22±98.39 to 176.45±91.45) (*p*-value <0.001). Melatonin also significantly reduced ALT, AST, total bilirubin, and direct and MELD scores in patients with liver disease (*p*-value <0.05).

**Conclusion:**

Melatonin may increase platelet count and inhibit thrombocytopenia in patients with liver disease; however, more investigations are needed to confirm the current results.

## Introduction

 Thrombocytopenia, as one of the complications caused by chronic liver disease (CLD), can be defined as a platelet count less than 150000 per μL (1). The prevalence rate of thrombocytopenia ranges widely from 6% to 78% in patients, depending on the type of liver disease (2-4). The liver plays a main role in the production and destruction of platelets. As reported in previous studies, there is a direct correlation between the severity of thrombocytopenia and the stage of liver disease (5).

Some mechanisms for thrombocytopenia induced by liver disease have been explained, such as a decrease in *Thrombopoietin* as an endogenous hormone released by liver, which plays a main role in this complication (6). A greater reduction in *thrombopoietin* production occurs when liver disease is exacerbated, like in a higher stage of fibrosis (7). Other causes of thrombocytopenia in liver disease include suppression of the bone marrow production of platelets due to inflammation and platelets being trapped in the spleen (1).

Melatonin is an endocrinic hormone secreted by different parts of body, such as the liver and the pineal gland, into the gastrointestinal system and plasma, respectively (8). Different therapeutic effects for melatonin have been observed, such as anti-oxidant and hepato-protective effects, regulation of the circadian rhythm, and the amelioration of side effects induced by chemotherapy (9). Moreover, the *Thrombopoietic* property of melatonin has been demonstrated in previous human studies (10, 11).

In their study, Lissoni et al. used 20 mg of melatonin as adjuvant therapy during chemotherapy in patients with cancer and reported a reduced prevalence of myelosuppression and thrombocytopenia (10).

According to pharmacological studies, melatonin is effective on thrombocytopenia associated with some cytokines such as IL-2, IL-12, TNF, and interferon alpha and stimulated megakaryocytes fragmentation into platelets (12, 11). 

Because of the lack of proper pharmacological agents for the treatment of thrombocytopenia in chronic liver disease, further clinical investigations are needed to propose a pharmacological therapy with less unpleasant side effects. This report is a part of the results of a randomized clinical trial (no. IRCT20180519039718N1) in which the anti-pruritic effect of melatonin in liver disease was evaluated. 

## Methods

This cross-over, double-blind, randomized, placebo-controlled trial was conducted on patients with pruritus induced by liver disease who referred to the Liver Disease Clinic affiliated with Tehran University of Medical Sciences (TUMS) in Tehran, Iran, from July 15, 2018 to January 31, 2019.

The RCT entitled “A Pilot Randomized Clinical Trial of the Anti-Pruritic Effect of Melatonin in Patients with Chronic Liver Disease” was accepted on April 21, 2020 in the Iranian Journal of Pharmaceutical Research (IJPR) and registered in the Iranian Registry of Clinical Trials (no. IRCT20180519039718N1) with ethics approval no. IR.TUMS.TIPS.REC.1397.043. 

In this RCT, the included participants were allocated into two groups receiving either melatonin-placebo or placebo-melatonin by computer–generated randomization in four block sizes (A, B, C, and D).

The patients were allocated into two groups that received either two 5-mg pearls (along with 10 mg at night orally) of melatonin (NutraLab Company, Canada, supplied by Zahravi Pharmaceutical Companies, Iran) or 2 pearls of a placebo (Zahravi Pharmaceutical Companies, Iran) for a 2-week period. Then, after a two-week washout period, patient groups were switched. None of the patients had received any new medicine which could have increased platelet count in the 4 weeks prior to being enrolled in this study.

Liver function tests, including alanine amino-transferase (ALT), aspartate aminotransferase (AST), alkaline phosphatase (ALP), total and direct bilirubin, serum creatinine (SrCr), international normalized ratio (INR) for calculating “model for end-stage liver disease” (MELD) score, and complete blood count (CBC) with differentiation and platelet count were assessed once at the beginning and once at the end of each treatment duration (laboratory data was evaluated 4 times). Eligible patients who had experienced pruritus induced by liver disease for at least 4 weeks were included in this study. The exclusion criteria were any history of hypersensitivity to melatonin, uncontrolled epilepsy, pregnancy, breastfeeding, decompensated liver disease, unstable hemodynamic conditions such as a mean arterial pressure <65 mmHg (13), or a chronic kidney disease with creatinine clearance <15 ml/min or dialysis (14). 

**Statistical analysis**

SPSS software (version 20.0, SPSS Inc., Chicago, IL, USA) was used for statistical analyses. Continuous data was reported as mean ± SD, non-normal values were presented by median (interquartile range), and categorical values were presented by frequency (percentage). To evaluate the normality distribution, the Kolmogorov–Smirnov test was performed on numerical variables. An independent t-test and Mann-Whitney U test were used to compare parametric and non-parametric variables, respectively. Cohen's d was calculated with a 95% confidence interval (95% CI) (15). A *p*-value < 0.05 was considered as statistically significant. 

## Results

In this study, 40 eligible patients were followed up until the end of the research. The demographic data of the patients are summarized in Table 1.

**Table 1:** Patient characteristics and diagnosis data at baseline

Group (n=40)	Melatonin-Placebo (n= 18)	Placebo-Melatonin (n= 22)	P-value^*^
Age, year, mean ± SD	41.5 ± 12.08	49.73 ± 12.89	0.2
Sex (F/M)	7/11	11/11	0.48
Etiology			
PSC, PBC, drug induced Liver disease (cholestatic)	10	13	
Cirrhosis (non-cholestatic)	8	9	
Baseline laboratory data, mean ±SD			
PLT *103/mm3	195.17 ± 104.65	168.41 ± 86.00	0.21
ALT, U/L	82.53 ±75.08	79.20 ± 75.57	0.84
AST, U/L	73.30 ±52.43	81.86 ± 63.87	0.57
ALP, U/L	738.64 ± 593.77	575.36 ± 431.44	0.15
Bilirubin Total, mg/dl, Median (Q1-Q3)	2.85 (1.32 -8.60)	1.3 (0.87-3.75)	0.01
Bilirubin direct, mg/dl, Median (Q1-Q3)	1.3(0.66-6.72)	0.48(0.3-1.75)	<0.05
INR, Median (Q1-Q3)	1.07 (0.93-2)	1.11 (1-2.7)	0.24
MELD	11.25 ± 6.04	9.93 ± 4.82	0.28

Abbreviations: primary biliary cholangitis (PBC), primary sclerosing cholangitis (PSC), Alanine transaminase (ALT), Aspartate transaminase (AST), Alkaline phosphatase (ALP), bilirubin (Bili), International normalized ratio (INR), Platelets (PLT), MELD Score (Model for End-Stage Liver Disease), number of patient (n). * p-value <0.05 is significant

**Figure 1:**
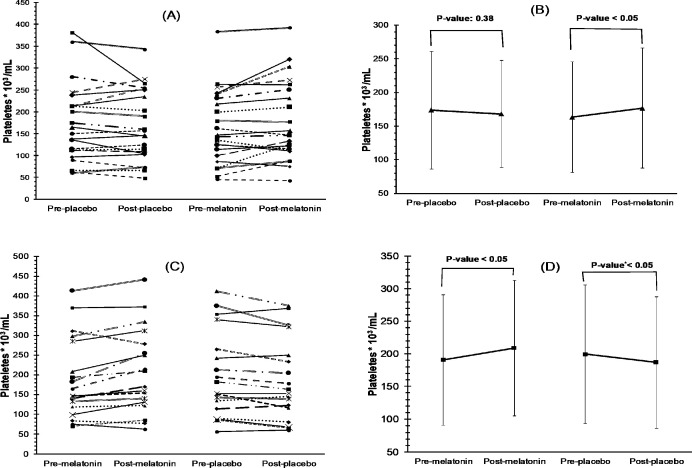
Trend of platelet counts of each patient at base and after 2 weeks of receiving placebo or melatonin and differences in average platelet count pre- and post-exposure to placebo and melatonin were shown for the Placebo-Melatonin and Melatonin-Placebo groups in A-B and C-D, respectively (A *p*-value<0.05 is significant). *p-value 0.03 due to negative effect

The platelet count was significantly elevated by melatonin compared to the placebo (*p*-value <0.05) (Table 2). Notably, the effect size (Cohen’s d) was 0.95 (CI 95%, 0.30_1.61). Moreover, 72.5% of 40 patients had an increase in platelet count, the average percent of which was 18.13% ± 18.74%, while 42.5% of 40 patients in the placebo group had an increase in their platelet count with an average of 7.45 ± 5.88 (*p*-value <0.05). Figures 1 and 2 show the trend of platelet count for each patient who received melatonin or the placebo, respectively.

**Table2:** Comparing melatonin and placebo effects on platelet and LFTs after 2 weeks

placebo	melatonin		
185.22±98.39	175.67±92.84	At baseline	PLT* 10^3^/mm^3^
176.45±91.45	191.10±98.82	After treatment	
8.77±25.80	-15.42±24.99	Difference between baseline and after treatment	
**0.03** ^†^	**<0.001**	p-value^*^	
77.07±72.33	84.32±78.11	At base line	ALT (U/L)
70.42±41.71	59.20±37.86	After treatment	
6.65±63.55	25.12±56.82	Difference of base and after treatment	
0.51	**0.008**	p-value^*^	
74.55±57.52	82.37±60.44	At base line	AST (U/L)
73.82±47.59	68.57±44.21	After treatment	
0.72±29.56	13.80±39.05	Difference of base and after treatment	
0.87	**0.03**	p-value^*^	
650.50±493.10	642.17±540.30	At baseline	ALP (U/L)
624.18±450.93	592.50±454.50	After treatment	
31.32±263.43	49.67±231.30	Difference between baseline and after treatment	
0.45	0.18	p-value^*^	
3.66±6.42	5.23±10.03	At baseline	Bilirubin Total (mg/dl)
3.85±6.17	4.22±7.59	After treatment	
-0.30±1.70	1.01±3.63	Difference between baseline and after treatment	
0.18	**0.01**	p-value^*^	
2.06±3.75	3.14±5.31	At baseline	Bilirubin Direct (mg/dl)
2.29±4.05	2.52±4.7	After treatment	
-0.22±1.14	0.61±2.15	Difference between baseline and after treatment	
0.21	**0.01**	p-value^*^	
1.25±0.40	1.29±0.37	At baseline	INR
1.23±0.35	1.24±0.38	After treatment	
0.01±0.20	0.05±0.24	Difference between baseline and after treatment	
0.80	0.15	p-value^*^	
0.88(0.24)	0.90(0.23)	At baseline	SCr mg/dl
0.89(0.23)	0.86(0.23)	After treatment	
-0.01(0.09)	0.03(0.13)	Difference between baseline and after treatment	
0.77	0.10	p-value^*^	
9.87±5.17	11.17±5.62	At baseline	MELD score
10.17±5.37	9.77±5.27	After treatment	
-0.30±1.69	1.40±2.89	Difference between baseline and after treatment	
0.27	**0.004**	p-value^*^	

Abbreviations: Alanine transaminase (ALT), Aspartate transaminase (AST), Alkaline phosphatase (ALP), International normalized ratio (INR), Liver function tests (LFTs), Platelets (PLT), Serum Creatinine (SCr), MELD Score (Model For End-Stage Liver Disease). All data are presented in mean±SD. P-value <0.05 is significant. * p-value for comparing patients’ difference of parameter after and before each phase exposure) † p-value 0.03 due to negative effect.

A significant reduction was also observed in the values of ALT, AST, total bilirubin, and direct and MELD scores in patients who received melatonin (*p*-value <0.05) (Table 1). Participants received no drugs or supplements which could affect the platelet count.

## Discussion

In this study, platelet count was significantly increased by melatonin consumption (from 175.67±92.84 to 191.10±98.82) compared to placebo (from 185.22±98.39 to 176.45±91.45) (*p*-value <0.001). The effect size (Cohen’s d) of 0.95 showed that melatonin had a large treatment effect in comparison with the placebo in patients with CLD. For the first time, this study has asserted melatonin’s positive effect on platelet count in chemotherapy-induced thrombocytopenia (16). In fact, according to a systematic review performed on the therapeutic effects of melatonin in cancer, melatonin may be considered for the treatment of chemotherapy-induced thrombocytopenia. Although the exact protective mechanism of melatonin against platelet destruction is still unknown, it is currently used as an adjuvant treatment during chemotherapy (16).

Paolo et al. studied a heterogenic population in radiation oncology or hematology and showed the *thrombopoietic* properties of melatonin. Moreover, they showed that the platelet count of 84% of 32 patients, who had thrombocytopenia related to either cirrhosis (7 patients) or liver metastases (25 patients) and then received 20 mg of melatonin for one month, reached the normal range of at least 100000 /μL, and the average percentage of increase in platelets was estimated to be 185%± 34% (11). The duration of treatment and dose of melatonin were both higher in Paolo’s study than in the current one; therefore, it seems that the higher dose and longer duration more efficiently improved platelet count. 

The response to *thrombopoietic* therapy is defined as an increase by more than 30000/ μL or doubling of the baseline count (17); however, in studies on liver disease-induced thrombocytopenia, such as Kalambokis et al., the *thrombopoietic* properties of rifaximin 1200 mg for one month in cirrhotic patients were reported as inhibitors of bacterial overgrowth. It was also shown that rifaximin significantly increased platelet counts in comparison with a placebo (88900 ± 37200 / μL to 109700± 39700/ μL vs. 76800 ± 30300/ μL to 81100± 28500 / μL, *p*-value <0.05) (18). 

The current, study demonstrated that melatonin decreased ALT (84.32±78.11 to 59.20±37.86), AST (82.37±60.44 to 68.57±44.21), direct bilirubin (3.14±5.31 to 2.52±4.71), total bilirubin (5.23±10.03 to 4.22±7.59), and MELD score (11.17±5.62 to 9.77±5.27), all of which were statistically significant (*p*-value <0.05). The current results reflected the findings of Chojnacki et al.’s placebo-controlled study, confirming that consumption of 5 mg of melatonin twice daily significantly decreased the elevated liver enzymes that were induced by statins (*p*-value <0.001) (19).

Some studies have also demonstrated that melatonin has some hepato-protective properties (20, 19).

Herein, it was hypothesized that alleviation of the severity of liver disease by the anti-fibrotic and antioxidant properties of melatonin may lead to the increased production of *thrombopoietin* by the liver. 

Melatonin significantly increased platelet counts in patients with liver disease compared to the placebo (*p*-value < 0.05). This study was not designed to evaluate the *thrombopoietic* properties of melatonin; therefore, it would be worthwhile to perform studies to determine the optimum dose of melatonin, onset of action, and duration of the *thrombopoietic* properties.
